# Targeting the mechano-microenvironment and liver cancer stem cells: a promising therapeutic strategy for liver cancer

**DOI:** 10.20892/j.issn.2095-3941.2023.0229

**Published:** 2023-11-24

**Authors:** Xiaorong Fu, Yi Zhang, Qing Luo, Yang Ju, Guanbin Song

**Affiliations:** 1 School of Biology and Engineering, Guizhou Medical University, Guiyang 550000, China; 2 College of Bioengineering, Chongqing University, Chongqing 400030, China; 3 Department of Mechanical Science and Engineering, Nagoya University, Nagoya 4648603, Japan

**Keywords:** Liver cancer, mechano-microenvironment, cancer stem cells, tumor heterogeneity, mechanotherapy

## Abstract

Over the past 2 decades, cancer stem cells (CSCs) have been identified as the root cause of cancer occurrence, progression, chemoradioresistance, recurrence, and metastasis. Targeting CSCs is a novel therapeutic strategy for cancer management and treatment. Liver cancer (LC) is a malignant disease that can endanger human health. Studies are increasingly suggesting that changes in the liver mechanical microenvironment are a primary driver triggering the occurrence and development of liver cancer. In this review, we summarize current understanding of the roles of the liver mechano-microenvironment and liver cancer stem cells (LCSCs) in liver cancer progression. We also discuss the relationship between the mechanical heterogeneity of liver cancer tissues and LCSC recruitment and metastasis. Finally, we highlight potential mechanosensitive molecules in LCSCs and mechanotherapy in liver cancer. Understanding the roles and regulatory mechanisms of the mechano-microenvironment and LCSCs may provide fundamental insights into liver cancer progression and aid in further development of novel therapeutic strategies.

## Introduction

Cancer is a major health-threatening disease and is the second leading cause of death worldwide^1,2^. Globally, liver cancer is among the most common cancers: approximately 900,000 new cases and more than 830,000 new deaths occurred in 2020, according to GLOBOCAN 2020, and liver cancer has become the second leading cause of cancer-related deaths after lung cancer^1,3^. In China, the incidence and mortality rate associated with liver cancer are high. Approximately 431,383 new liver cancer cases, and approximately 412,216 new deaths were estimated to have occurred in China in 2022, accounting for approximately half of all new liver cancer patients and deaths worldwide^1,4^. Poor treatment efficacy, high rates of recurrence and metastasis, and low survival rates of liver cancer pose a heavy economic burden on families and society. Therefore, exploring effective new methods to treat liver cancer remains a major challenge for clinical experts.

The liver, performs several important functions and has a remarkable ability to regenerate itself. However, illnesses can cause permanent and irreversible liver damage. Liver tissue mechanical properties substantially change during pathogenesis. Tissue stiffness increases from normal liver tissue to fibrotic liver tissue, cirrhotic liver tissue, and hepatocellular carcinoma (HCC) liver tissue; the stiffness of cancerous liver tissue is approximately 10-fold higher than that of normal liver tissue^5–8^. In addition, liver cancer tissue shows mechanical heterogeneity, wherein the core is softest, the greatest stiffness is observed at the invasive external edges, and the middle region has intermediate stiffness^6^. Studies are increasingly suggesting that changes in the liver mechanical microenvironment during liver tissue pathogenesis are a primary driver triggering the occurrence and development of liver cancer^9–11^.

Recently, a small subpopulation of cells sharing features with somatic stem cells, referred to as cancer stem cells (CSCs), have been identified in most tumors and demonstrated to be the root cause of cancer occurrence, progression, chemoradioresistance, recurrence, and metastasis^12^. Liver cancer stem cells (LCSCs) are responsible for tumor growth, recurrence, and metastasis in HCC, as well as failure of chemotherapy and radiotherapy^13^. Targeting liver LCSCs may be an emerging approach for diagnosing and treating liver cancer. Therefore, in this review, we discuss the crucial roles of the liver mechano-microenvironment and LCSCs in the progression of liver cancer, and the relationship between the mechano-microenvironment and LCSCs, to provide insights into new therapeutic approaches.

## The mechanical microenvironment and liver cancer progression

A healthy liver comprises 2 main lobes, consisting of 8 segments and thousands of lobules. These lobules are connected to small ducts, which in turn are connected to larger ducts forming a common hepatic duct. Blood flows through the larger ducts to the small ducts, and then to the lobules, which consist primarily of hepatocytes and sinusoids. The sinusoid is a discontinuous capillary whose caliber is larger than that of other capillaries, and it has a lining of specialized endothelial cells called liver sinusoidal endothelial cells (LSECs). Kupffer cells are present in the sinusoids and have clearance functions^14,15^. The space of Disse between the hepatocytes and sinusoids contains a low density of extracellular matrix (ECM) and quiescent hepatic stellate cells (HSCs)^16^.

The liver performs vital functions such as synthesis, nutrient processing, and detoxification, and liver failure is a critical health threat. Hepatitis is a common liver disease, and many patients with liver cancer progress sequentially from the onset of chronic liver diseases such as hepatitis, fibrosis, cirrhosis, and HCC^17^. Furthermore, tissue stiffness increases from normal liver tissue to liver fibrosis, cirrhosis, and HCC; the stiffness of liver cancer tissue has been reported to be approximately 10-fold that of normal tissue^5–8^. Increased cancer cell density and size are among the most important contributors to the enhanced stiffness in HCC tissue. In this section, we describe the relationship between mechanical changes in liver disease and the development of liver cancer, as well as the mechanical heterogeneity of the liver intratumor tissue.

### Changes in mechanical properties during liver tissue canceration

Chronic liver diseases, such as chronic viral hepatitis caused by hepatitis B or C viral infection, metabolic dysfunction-associated steatotic liver disease, and alcoholic liver disease, can damage the liver. The development of liver fibrosis is based on chronic, recurrent, and persistent liver damage, and is a pathological phenomenon that occurs during the wound-healing response to liver tissue damage. During this process, collagen, glycoproteins, proteoglycans, and other ECM molecules are overproduced and abnormally distributed in the liver; the rate of production exceeds the rate of degradation, thereby resulting in ECM deposition and causing abnormalities in liver structure and function^18,19^, and consequently contributing to liver stiffening. Liver fibrosis is a pre-cirrhotic lesion that serves as the basis for cirrhosis development.

**Figure 1:**
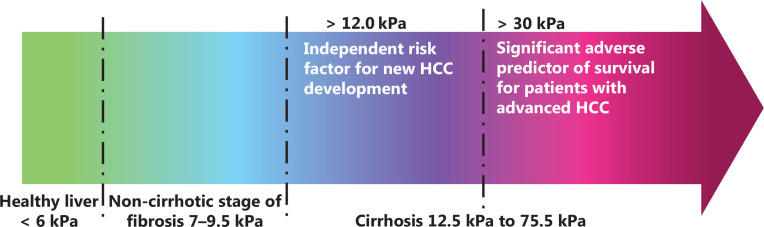
Pathological changes in liver tissue stiffness, detected by ultrasound transient elastography. The tissue stiffness increases from normal liver tissue to liver fibrosis and cirrhosis. The stiffness of normal liver tissue is below 6 kPa^20^. The stiffness of the non-cirrhotic stage of fibrosis (F1–F3) is 7–9.5 kPa^8^. The stiffness of F4 fibrosis (cirrhosis) is > 12.5 kPa^20^. The range of liver cirrhosis stiffness is similar to that of liver cancer tissue. Liver stiffness > 12.0 kPa is an independent risk factor for new HCC development^10^. Liver cancer tissue > 30 kPa is a significant adverse predictor of survival in patients with advanced HCC^21^.

According to the METAVIR classification system, liver fibrosis can be classified into 4 stages: F0–F1 (absent or mild fibrosis), F2 (significant fibrosis), F3 (severe fibrosis), and F4 (cirrhosis)^5^. Stiffness gradually increases from normal liver tissues to multi-stage liver fibrosis, liver cirrhosis, and HCC^5^. Liver stiffness, measured with ultrasound-based transient elastography (Fibroscan technique) ranges from 2.5 to 7 kPa in the F0 to F1 stage, to 7.1 kPa in the F2 stage, 9.5 kPa in the F3 stage, and 12.5 to 75.5 kPa in the F4 stage^8^. The highest degree of liver cirrhosis can reach 75.5 kPa, similar to the range of liver cancer tissue stiffness^7^. The liver tumor stiffness, measured with ultrasound transient elastography, is 55 kPa in HCC, 75 kPa in cholangiocellular carcinoma, and 66.5 kPa in metastatic tumors^7^. Mueller et al.^20^ have also used ultrasound transient elastography to measure liver stiffness and have concluded that the stiffness of normal liver tissue is below 6 kPa, that of F3 fibrosis is 8 kPa, and that of F4 fibrosis (cirrhosis) is 12.5 kPa. Tatsumi et al.^10^ have analyzed 1,002 cases (246 with HCC and 756 without HCC) and investigated the fibrotic stage at which the risk of HCC development increases; they have reported that liver stiffness > 12.0 kPa is an independent risk factor for HCC development. A recent study has indicated that stiffness > 30 kPa (detected *via* transient elastography) is a significant adverse predictor of survival in patients with advanced HCC^21^. Transient elastography has been reported to have several limitations^22,23^. Currently, atomic force microscopy (AFM) is used to detect liver tissue stiffness. Transient elastography can measure only the bulk stiffness of targeted tissue, whereas AFM can measure local tissue stiffness quantitatively. Normal liver tissue stiffness, as detected by AFM, is approximately 150 Pa, whereas in fibrotic liver tissue, the stiffness ranges from 1 to 6 kPa^24^. Sun et al.^6^ have used N-nitrosomorpholine to stimulate the development of hepatitis, fibrosis, early stage cirrhosis, late-stage cirrhosis, and HCC, and measured the stiffness of each group by AFM. The stiffness of control samples (normal liver tissue) was 0.113 kPa; that in the hepatitis group without collagen proliferation was 0.19 kPa; that in the liver fibrosis group increased to 0.245 kPa; that in the early-cirrhotic group increased to 0.344 kPa; that in the late-cirrhotic group significantly increased to 0.79 kPa; and that in the HCC group reached 1.65 kPa^6^. The above findings indicate that both transient elastography (**Figure 1**) and AFM measurements show an increasing trend of stiffness from normal liver tissue to fibrous liver tissue and finally to liver cirrhosis/liver cancer tissue (**Table 1**).

**Table 1:** Pathological changes in liver tissue stiffness

Species	Healthy	Non-cirrhotic stage of fibrosis (F1–F3)	Cirrhosis (F4)	HCC	Detected location	Methods	References
Human	2.5 kPa	7–9.5 kPa	12.5–75.5 kPa		Bulk stiffness of targeted tissue	Transient elastography	^8^
Human				55 kPa	Bulk stiffness of targeted tissue	Transient elastography	^7^
Human	< 6 kPa	8 kPa	12.5 kPa		Bulk stiffness of targeted tissue	Transient elastography	^20^
Human				> 12.0 kPa	Bulk stiffness of targeted tissue	Transient elastography	^10^
Mouse	150 Pa	1–6 kPa			Quantitative local tissue stiffness	Atomic force microscopy	^24^
Rat	0.113 kPa	0.19–0.245 kPa	0.344–0.79 kPa	1.65 kPa	Quantitative local tissue stiffness	Atomic force microscopy	^6^

**Figure 2:**
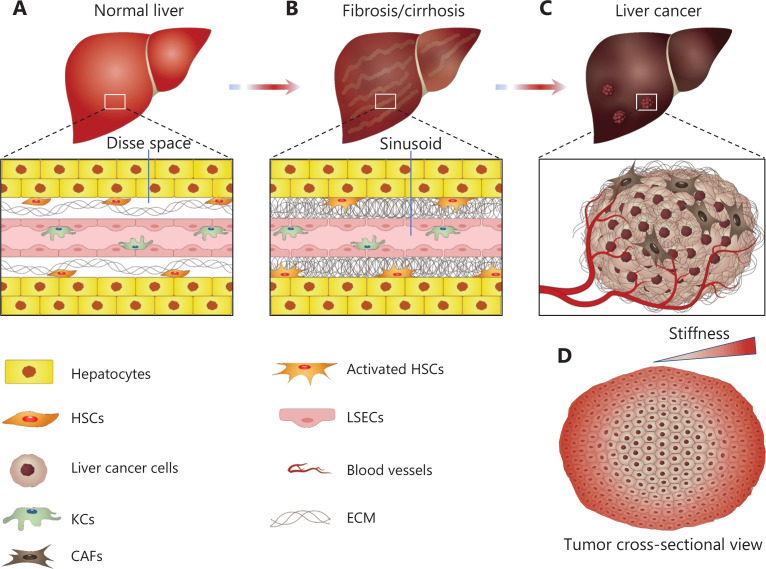
The mechanical microenvironment of the liver is closely associated with pathological changes in the liver. (A) Hepatocytes and LSECs are separated by the space of Disse, which contains minimal ECM in healthy liver tissue. Quiescent HSCs reside in the space of Disse. Kupffer cells exist in the sinusoid. (B) In liver fibrosis/cirrhosis, HSCs are activated and contribute to the production and deposition of ECM, thereby contributing to stiffening. (C) Liver cirrhosis with elevated stiffness is strongly associated with the development of liver cancer. Quiescent HSCs and other cell types are transformed into CAFs, which produce large amounts of ECM deposition around liver tumors. ECM deposition and vascular penetration contribute to increased stiffness and mechanical heterogeneity in liver tumors. (D) Liver cancer tissue displays mechanical heterogeneity, gradually increasing in stiffness from the core to the invasive periphery.

A direct relationship exists between cirrhosis and the development of liver cancer. Cirrhosis is the main cause of precancerous lesions of liver cancer. Reports have indicated that 80%–90% of HCC cases occur because of liver cirrhosis^25,26^, and liver cirrhosis is a unifying risk factor for the progression of HCC^9–11^. Liver cancer tissue is approximately 10-fold stiffer than normal liver tissue^5–8^. Therefore, investigating the relationship between the changing stiffness under liver pathological processes and the progression of liver cancer may be an emerging approach for improving understanding of liver cancer biology and cure (**Figure 2A–2C**).

### Mechanical heterogeneity of liver cancer tissue

The heterogeneity of liver cancer poses serious challenges for developing and controlling systemic therapies. Liver cancer displays mechanical, cellular, molecular, and metabolic heterogeneity. In this section, we discuss the mechanical heterogeneity of liver cancer tissues (**Figure 2C, 2D**). The stiffness of liver tissue in different intratumor regions varies. In liver cancer tissues, the core part of the tumor is softest, the invasive front tissue is stiffest, and the middle part has intermediate stiffness^6,27,28^. Breast intratumor tissues have also been reported to exhibit mechanical heterogeneity. The core area of the breast intratumor tissue is softest, the middle area has intermediate stiffness, and the invasive peripheral area is stiffest^29,30^. Both liver and breast intratumor regions show mechanical heterogeneity. However, only a few reports on mechanical heterogeneity in the intratumor regions of other types of cancer tissues have been published. Further studies are required to determine the mechanical heterogeneity of cancerous tissues.

Understanding the reason for the mechanical heterogeneity of liver cancer tissues is essential for improving understanding of cancer biology. The rapid proliferation of liver cancer cells results in the formation of liver cancer tissues consisting of a core region of cancer cells and an invasive front region (**Figure 2D**). A recent study has identified and characterized a 500 μm-wide invasive zone centered on the tumor border in liver cancer, which was defined as the region within 250 μm on both sides (tumor tissue side and paratumor tissue side) of the tumor margin areas (the border); enrichment of fibroblasts on the tumor side of the border compared with the paratumor side has been observed^31^. Highly activated fibroblasts in metastatic cancer increase stiffness and are a major source of cancer-associated fibroblasts (CAFs)^32^. CAFs, the principal producers of ECM in solid tumors^33,34^, lead to substantial deposition of ECM surrounding tumors (**Figure 2C**). The specific microenvironment of cancer tissues also stimulates quiescent HSCs to activate CAFs^35^. Furthermore, studies have shown that CAFs express lysyl oxidase (LOX), thereby initiating collagen crosslinking^36,37^. The abundant deposition of ECM and crosslinking at the external invasion site increases ECM stiffness, which in turn enhances cancer cell tension^27,38^ in the invasive front of liver cancer tissues. However, ECM deposition gradually decreases from the invasive front to the core^6^. Therefore, outside the invasive front of cancer tissue, ECM deposition is affected by compressive pressure resulting from the spatial occupancy effect of the rapidly proliferating cancer cells and the massive deposition of ECM. Simultaneously, ECM crosslinking further increases ECM stiffness, and the cell-ECM connections and ECM stiffening increase the cell tension^27,39^. Furthermore, cancer tissue located at the invasive front is constrained by the surrounding normal tissue, and consequently is subjected to the stretching force of normal tissues. However, the cancer tissue core is affected primarily by compressive pressure resulting from the spatial occupancy effect of the rapid proliferation of cancer cells. All the above mechanical regimes together contribute to greater solid stress at the invasive front of cancer tissues than at the core; consequently, the invasive front is much stiffer than the core.

In addition, vascular permeability in cancer tissues squeezes the surrounding tissues (**Figure 2C**) and indirectly increases cancer tissue stiffness. High vascular permeability is key in increasing interstitial fluid pressure (IFP)^40,41^. Intravascular fluid and solutes leave the capillaries under blood pressure and enter the interstitial microenvironment of cancer tissues, thereby increasing hydrostatic interstitial fluid pressure^38^. The external parts of tumors are more vascularized, whereas the internal supply of oxygen and nutrients becomes insufficient because of a lack of blood vessel distribution, thus leading to internal cancer cell necrosis^42,43^. Necrosis at the core of HCC, which generally occurs with tumor growth, is an important reason for the lower stiffness in the core of HCC^44^. Lower vascular distribution may be another reason for the lower internal stiffness than the external marginal stiffness of cancer tissue. Mechanical heterogeneity at different sites in cancer tissue may lead to heterogeneity in cellular functions. The mechanical heterogeneity of liver cancer tissue, and its influence on cancer cell behavior, warrants further in-depth study.

In summary, pathological mechanical changes in liver tissue play important roles in the occurrence and development of liver cancer (**Figure 2A–2C**). In addition, liver intratumor regions display mechanical heterogeneity (**Figure 2D**). Targeting the mechanical microenvironment may enable breakthroughs in the understanding and treatment of liver cancer.

## LCSCs and liver cancer progression

**Figure 3:**
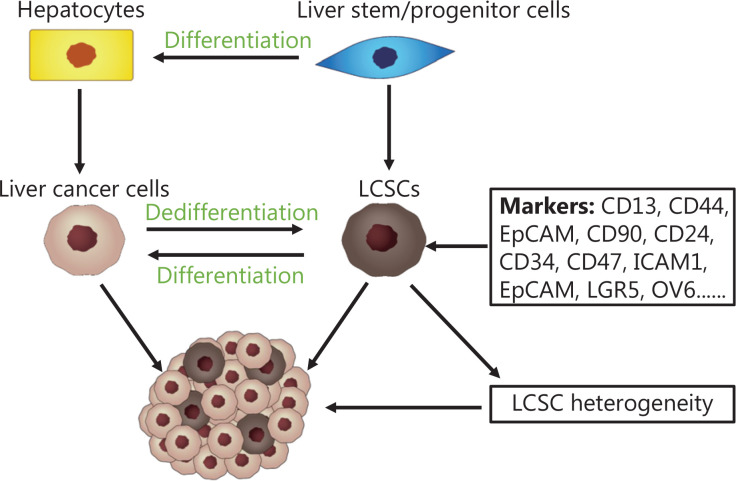
Potential origin and heterogeneity of LCSCs. LCSCs may originate from non-LCSCs that undergo dedifferentiation and share many similar characteristics with normal stem cells. LCSC heterogeneity in different subpopulations is identified by the expression of various LCSC markers. Different LCSC subpopulations are independently associated with the prognosis of HCC and contribute to intratumor heterogeneity and tumor progression.

**Figure 4:**
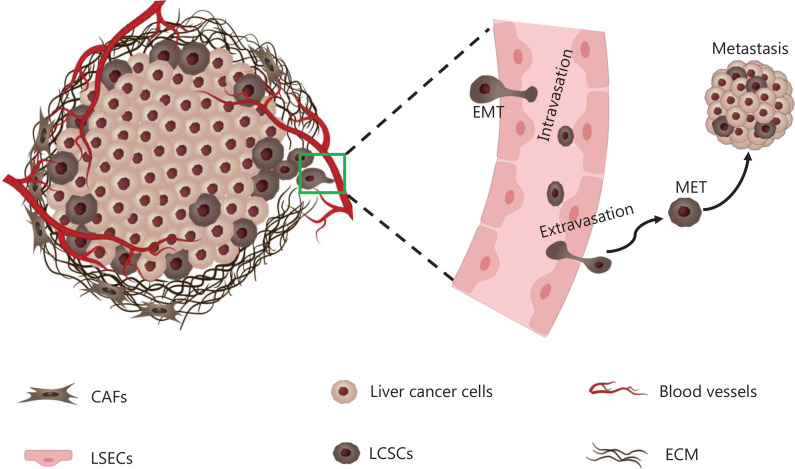
Relationships among mechanical heterogeneity, LCSCs, and EMT. The distribution of LCSCs within liver cancer tissue is heterogeneous and preferentially distributed in the stiffer invasive front. The stiffer invasive front of liver cancer tissue facilitates EMT in LCSCs, thus resulting in cancer tissue invasion, dissemination, and growth at metastatic sites.

Studies are increasingly indicating that, in liver cancer cells, a small subpopulation of cell types, named LCSCs, have the capacity for self-renewal, chemotherapy resistance, recurrence, and metastasis^13^. Furthermore, the distribution and stemness of LCSCs are affected by the mechanical heterogeneity of the liver intratumor region^6^. In this section, we focus on the roles of LCSCs in the progression and metastasis of liver cancer, and on how the mechanical heterogeneity of the liver intratumor region influences LCSC distribution, stemness, and function (**Figures 3 and 4**).

### Characteristics of LCSCs

The liver is a unique organ exhibiting remarkable plasticity and regenerative potential after injury. Liver regeneration may involve the activation of fully differentiated hepatocytes or liver stem/progenitor cells. Hepatocytes are responsible primarily for maintaining the liver under normal conditions and responding to acute liver injury. In severe liver diseases, such as hepatitis and liver fibrosis/cirrhosis, hepatocyte replication potential is impaired, and liver stem/progenitor cells can be activated to supply new hepatocytes and maintain the functional integrity of the liver^45^. Hepatocytes are the cells of origin in HCC^46^. Transformed hepatocytes can also activate liver stem/progenitor cell proliferation and promote tumor heterogeneity during the early stages of HCC^47^. In addition, liver cancer progenitor cells exist in precancerous areas and contain foci of mutated hepatocytes showing CSC-like features; therefore, liver cancer progenitor cells are considered the precursors of LCSCs^48^. Many LCSC markers found in HCC are oncofetal markers shared by hepatoblasts and liver stem/progenitor cells^47^. LCSCs have been inferred to originate from liver stem/progenitor cells, because they share many characteristics with normal stem cells. Furthermore, LCSCs are highly plastic. Transformed marker-negative liver cancer cells can acquire features of LCSCs, which in turn can differentiate into normal liver cancer cells^49–51^. Therefore, improved understanding of LCSC plasticity may help develop new LCSC-targeted therapies.

Various cell surface markers have been used to identify LCSCs in liver cancer. Nearly 20 molecular markers have been identified to isolate and characterize LCSCs^52^. Other studies have summarized LCSC surface markers in detail^13,52–55^. Surface markers, such as CD13, CD44, EpCAM, CD90, CD24, CD34, CD47, ICAM1, EpCAM, LGR5, and OV6, are widely recognized as markers of LCSCs, because of their association with proliferative capacity, self-renewal capacity, drug resistance, and recurrence of liver cancer. However, no specific markers specifically identify LCSCs, and simultaneous expression of more markers in LCSCs has been associated with greater stemness^56^. For example, the CD133^+^/ALDH^+^ subgroup of LCSCs displays stronger tumorigenicity than the CD133^−^/ALDH^+^, CD133^+^/ALDH^−^, or CD133^−^/ALDH^−^ subgroup of LCSCs, both *in vivo* and *in vitro*^57,58^. The CD133^+^/EpCAM^+^ subgroup of LCSCs shows higher tumor-initiating activity than the CD133^+^/EpCAM^−^ and CD133^−^/EpCAM^+^ subgroups of LCSCs^59^. LCSC heterogeneity in different subgroups is identified *via* expressed LCSC markers^56^. Different LCSC subgroups are independently associated with HCC prognosis and contribute to intratumor heterogeneity and tumor progression^60^. LCSC subgroups identified with single-cell surface markers have higher self-renewal ability than marker-negative cells, but LCSCs identified with different single-cell surface markers and different numbers of single-cell surface markers display appreciable biological differences^56^. LCSCs, defined by various cell surface markers, may contain different oncogenic drivers, thus posing challenges in identifying targeted therapeutic approaches. Because many markers have been discovered for LCSCs, and the antibody cost and time required can be substantial, many studies usually select only one or several markers as criteria to screen cell subpopulations of LCSCs. However, whether the undetected markers might affect the molecular mechanism of the detected marker-associated signaling pathways is unclear. Furthermore, comprehensive studies of different markers to discover their differences and associations in the regulation of liver cancer metastasis are lacking. In addition, whether new markers might better represent the LCSC subpopulation warrants further exploration (**Figure 3**).

### Implications of mechanical heterogeneity, LCSCs, and epithelial-mesenchymal transition (EMT)

As discussed above, both liver and breast cancer tissues show mechanical heterogeneity, wherein the invasive front is stiffest, and the core is softest. A recent study has indicated that the distribution of LCSCs is closely associated with the mechanical heterogeneity of liver cancer (**Figure 4**). Sun et al.^6^ have demonstrated that the greatest number of LCSCs (EPCAM^+^, CD13^+^, and CD44^+^) is found in the stiffer invasive front of the liver cancer tissue in a rat liver cancer model, thus indicating that stiffer liver cancer tissue (marginal part) is more suitable for potentiating the stemness of LCSCs. CSCs in breast cancer and glioma also have similar heterogeneity in distribution. CD24^−^CD44^+^ BCSCs are localized at the tumor-invasive front, whereas ALDH-expressing BCSCs are located more centrally^61^. In addition, glioma stem cells are preferentially located at the stiffer invasive front rather than at the softer intratumor core^62^. These findings suggest that the stemness maintenance of CSCs is location-dependent within cancerous tissues and is likely to be affected by the mechanical heterogeneity of cancer tissues. A stiffer invasive front may favor the distribution of CSCs because the mechanical microenvironment of the invasive front is more suitable for stemness maintenance and functions of CSCs.

EMT refers to epithelial cells that lose cell-to-cell connections, undergoes cytoskeletal remodeling leading to loss of polarity, and finally acquires a mesenchymal phenotype^63^. Furthermore, EMT is reversible, and the epithelial phenotype produced by mesenchymal-epithelial transformation (MET) is characterized by E-cadherin expression and the establishment of cell polarity. Cancer cells undergoing EMT have aggressive properties, such as metastasis, invasion, immune suppression, and drug resistance. Emerging studies indicate that EMT is an important contributor to CSC function in many cancer types, including liver cancer^64–67^. Cancer cells undergoing EMT have been reported to possess many stem cell-like features, such as elevated CD44 expression and enhanced ability to form spheroids^68^. CD24^−^CD44^+^ BCSCs are primarily quiescent and exhibit EMT phenotypes, whereas ALDH-expressing BCSCs are proliferative and exhibit the MET phenotype^61^. In squamous cell carcinoma, CSCs termed CD44^high^ESA^low^ have been found to be migratory and to have mesenchymal traits characteristic of EMT CSCs. In contrast, CSCs termed CD44^high^ESA^high^ are proliferative and retain epithelial characteristics (MET CSCs)^64^. CSCs associated with invasion and migration abilities have been associated with EMT, whereas CSCs associated with proliferation have been associated with MET. Furthermore, a study has revealed that EMT preferentially occurs in the stiffer invasive front of breast tumors in humans and mice^29^. Interestingly, CSCs are also preferentially located at the invasive front of tumors^6,61,69^. Therefore, the stiffer mechanical microenvironment of the invasive front may facilitate CSC EMT and consequently result in cancer tissue invasion, dissemination, and growth at metastatic sites (**Figure 4**). However, studies on the relationships among cancer tissue mechanical heterogeneity, CSC distribution, and EMT are lacking. Further studies focusing on this aspect may enhance understanding of the mechanical heterogeneity of cancer and CSC biology.

## Liver cancer therapy targeting the mechanical microenvironment and LCSCs

**Figure 5:**
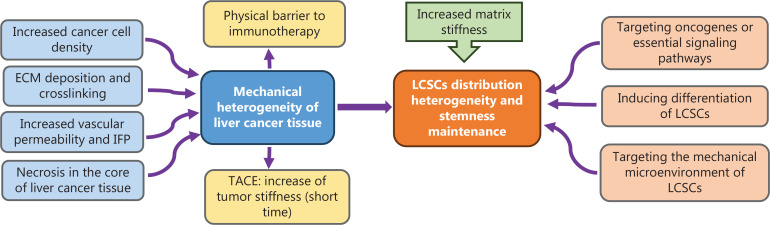
Liver cancer therapy targeting the mechanical microenvironment and LCSCs. The increased cancer cell density, ECM deposition and crosslinking, increased vascular permeability and IFP, and necrosis in the core of liver cancer tissue contribute to the mechanical heterogeneity of liver cancer tissue. Common HCC therapeutic strategies, such as TACE and immunotherapy, are associated with tumor stiffness. The distribution of LCSCs in liver cancer tissue is affected by the mechanical heterogeneity of liver cancer tissue. Increased matrix stiffness contributes to LCSC stemness maintenance. Focusing on the mechanical microenvironment and LCSCs in liver carcinogenesis may provide theoretical guidance for preventing and treating liver cancer in clinical settings.

The human body is continually exposed to various physical forces. These physical forces are called mechanotherapy in the context of rehabilitation and therapeutic outcomes^70^. Mechanotherapy was first defined in the Oxford English Dictionary in 1890 as “the treatment of disease by mechanical means”. Mechanotherapy is currently used as a physical therapy. Traditional physical therapy focuses primarily on rehabilitation, namely exercise therapeutics for injured tissues of patients recovering from trauma or surgery. Recent developments in mechanobiology have shed light on the effects of physical forces on cells and tissues^71^. Cells can be exposed to various micromechanical stimuli, including tension, compression, shear stress, and hydrostatic pressure, all of which affect their functions. However, mechanotherapy is seldom used as a therapeutic intervention for specific diseases such as cancer. Solid cancer tissue is typically much stiffer than the surrounding healthy tissues. In particular, liver cancer tissues usually present mechanical heterogeneity. Therefore, the use of mechanotherapy to explore how cancer cells in liver cancer tissue are affected by the mechanical microenvironment, as well as treatment of liver cancer through mechanical means, may greatly aid in improving the therapeutic approach for liver cancer. Moreover, the distribution and maintenance of LCSC stemness, which plays important roles in the progression and metastasis of liver cancer, are influenced by the mechanical heterogeneity of liver cancer tissues^6^. Therefore, in this section, we focus on the importance of targeting the mechanical microenvironment and LCSCs to improve liver cancer treatment (**Figure 5**).

### Targeting the liver cancer mechanical microenvironment

#### Targeting the ECM

In the liver tumor tissue microenvironment, with the rapid proliferation of cancer cells, an increase in the number of non-cancer cells, and overproduction and structural remodeling of the ECM, the tumor microenvironment (TME) density gradually increases and enhances stiffness. In addition, vascular penetration contributes to increased stiffness. ECM stiffening upregulates exosome secretion, which in turn leads to changes in the TME that promote tumor growth^72^. Increased matrix stiffness induces upregulation of Piezo1, which restrains ubiquitination of the hypoxia-inducible factor-1α (HIF-1α) and subsequently enhances the expression of downstream pro-angiogenic factors, thereby accelerating HCC angiogenesis^73^. Therefore, blocking ECM deposition and angiogenesis can downregulate tumor stiffening. CAFs are the primary donors of massive ECM deposition around tumors^33,34^, and ECM stiffening contributes to angiogenesis^74^. One study has indicated that downregulating liver metastasis stiffness by inhibiting the activity of metastasis-associated fibroblasts with renin-angiotensin system (anti-RAS) inhibitors decreases ECM stiffness, which in turn increases the anti-angiogenic effects of bevacizumab in patients with metastatic colorectal cancer^74^. The main components of the ECM are collagen and matrix metalloproteinases (MMPs), which are enzymes that specifically degrade collagen. One study has cultured HCC cells in a collagen-based gel tailored with an elastic modulus of ≈4.0 kPa. Subsequently, the gel was treated with MMP-1 to decrease the elastic modulus from 4.0 to 0.5 kPa, thus resulting in greater HCC cell sensitivity to radiation than observed in cells grown on stiffer gel not exposed to MMP-1 treatment^75^.

#### Targeting the IFP

The IFP in most solid tumors is significantly higher than that in normal tissues^41,76^. Increased IFP in tumors forms a physiological barrier to drug delivery^77^. In a rabbit liver tumor model, the downregulation of IFP levels has been found to increase drug penetration into liver tumors^78^. High vascular permeability has been reported to be key in increasing IFP^40,41^. Inhibiting vascular permeability decreases IFP and upregulates anticancer drug delivery to tumors^79^. Furthermore, nanosystems loaded with anticancer drugs modified with cell-penetrating peptides readily penetrate tumor masses. Cell-penetrating peptides have high tissue penetration ability and low cytotoxicity, thereby overcoming the problems of low delivery efficiency and limited tumor-penetrating ability of drug carriers. Chen et al.^80^ have found that a cell-penetrating peptide (tLyP-1)-conjugated ZIF-90 nanosystem loaded with anticancer drugs penetrates the liver tumor mass with the help of tLyP-1, and can eradicate differentiated liver cancer cells and LCSCs simultaneously. The lipid-modified cell-penetrating peptide aggregates into micelles and enables simultaneous loading of the natural product narciclasine and ULK1 siRNA, which target HCC by synergistically inhibiting autophagy and inducing apoptosis^81^.

#### Liver cancer tissue stiffness and common HCC therapeutic strategies

Transarterial chemoembolization (TACE) indicates that chemotherapy drugs can be transported to local liver tumors through the blood vessels; simultaneously, the tumor’s blood vessels can be blocked with iodized oil, polyvinyl alcohol particles, or drug-loaded microspheres, thus decreasing or even cutting off the nutrient supply to tumors, and shrinking tumor size, delaying tumor progression, and extending survival time. TACE aggravates fibrosis and increases the stiffness of liver cancer tissues within short time periods^82,83^. Haas et al.^84^ have demonstrated that the size of liver tumors decreases after treatment with TACE, but the stiffness increases. Praktjknjo et al.^85^ have also shown greater stiffness in liver cancer tissue 3 days after TACE treatment than in non-treated liver cancer tissue. Hou et al.^86^ have analyzed 230 patients who underwent TACE and found that the tumor stiffness increased from 40.1 kPa (before TACE) to 60.4 kPa (after TACE), whereas 1 month after TACE, the stiffness decreased to 19.3 kPa. These results suggest that TACE therapy affects the mechanical microenvironment of liver cancer tissue, and decreased tumor size and increased tumor stiffness (during short time periods) are considered good responses^86^.

Tumor immunotherapy, one of the most successful methods in cancer therapy in recent years, is divided primarily into 2 types: cellular immunotherapy and immune checkpoint inhibitor (ICI) therapy. Cellular immunotherapy refers to collecting immune cells from the patient’s body and modifying them outside the body to achieve more effective and accurate immune activity against cancer cells. After the modified immune cells are injected back into the patient’s body, they are directed to kill cancer cells. Immune cells (such as T cells, B cells, dendritic cells, and mononuclear macrophages) are the primary cellular components of the tumor immune microenvironment^87^. The use of T-cells to specifically recognize and kill cancer cells has become a commonly topic in immune-based cancer therapies. However, in most immunotherapies for solid tumors, T cells are gradually depleted^88^. The TME is the main factor affecting normal T cell function^89^. Although most immune cells are suspension cells that exist in blood vessels, they must infiltrate the TME to kill tumor cells. Immune cells are inevitably affected by the mechanical microenvironment of tumors. Study has shown that the hardness of tumor tissues prevents immune cells from penetrating the TME^90^. Liu et al.^91^ have analyzed existing single-cell tumor sequencing results and found significant differences in gene expression in T cells from tumors, healthy tissues, and blood vessels. When T cells are exposed to collagen environments with high viscoelasticity, they tend to become memory T cells capable of killing tumor cells and display greater tumor size control in mice^91^.

ICI therapy involves immune cells that produce small protein molecules that inhibit their own functions. Tumor cells use this mechanism to suppress immune cells and survive by escaping the immune system. ICI eliminate this immune tolerance and allow immune cells to reactivate and kill cancer cells. However, the unique mechanical microenvironment of tumors is a major obstacle to ICI therapy. Tumor stiffening and solid stress elevation induce hypoperfusion and hypoxia by compressing tumor vessels^92^. Hypoperfusion and a stiff TME are physical barriers to T-cell infiltration into tumors^93^, whereas higher perfusion levels increase ICI therapy efficacy^94,95^. Downregulation of tumor stiffness has been found to enhance perfusion and ICI efficacy^92^. These results suggest that common HCC therapeutic strategies such as TACE and immunotherapy are closely associated with the tumor mechanical microenvironment. The mechanical microenvironment is a non-negligible factor in HCC treatment.

### Targeting LCSCs

#### Targeting oncogenes or oncoproteins and essential signaling pathways

LCSCs are believed to be at the origin of tumor recurrence, resistance, and metastasis. Therefore, drugs specifically targeting LCSCs must be developed^96^. Furthermore, stemness maintenance and functions of LCSCs are associated with oncogenes, oncoproteins, and signaling pathways. For example, YAP1 signaling initiated by long noncoding RNA drives the self-renewal and maintenance of CD13^+^ CD133^+^ LCSCs^97^. The TGF-β/H19 signaling axis, regulated by Sox2, in CD44^+^ LCSCs is important in promoting hepatocarcinogenesis^98^. In a mouse model of HCC, tumors with co-activation of both Akt/mTOR and Wnt/β-catenin signaling pathways have been found to contain a subpopulation of SP/CD44^+^ LCSCs, whose self-renewal and tumorigenesis are regulated by activated Stat3 pathways^99^. Src-homology 2 domain-containing phosphatase 2 (Shp2) facilitates LCSC (CD133^+^EpCAM^+^ subgroup) expansion by promoting the dedifferentiation of hepatoma cells and enhancing the self-renewal of LCSCs *via* amplifying β-catenin signaling^51^. The expression of inducible nitric oxide synthase in CD24^+^CD133^+^ LCSCs, but not CD24^−^CD133^−^ LCSCs, has been found to promote stemness characteristics of LCSCs *via* Notch1 signaling, and to accelerate HCC initiation and tumor formation in a mouse xenograft tumor model^100^. LGR5^+^ cells (LCSCs) are enriched in mouse liver tumors and are considered as an important LCSC subpopulation that contributes to organoid initiation, tumor formation, and chemotherapy resistance^101^. Lysine-specific demethylase 1 (LSD1) promotes activation of the β-catenin pathway, thereby maintaining the stemness and chemoresistance of the LGR5^+^ subgroup of LCSCs^101^. Moreover, liver cancer and many other malignant cancers exhibit “aerobic glycolysis”^102^. However, increased oxidative phosphorylation is required for the stemness maintenance of LCSCs^103^. Wei et al.^104^ have revealed that LCSCs rely on enhanced mitochondrial respiration to maintain their stemness properties, in contrast to aerobic glycolysis, which plays a major role in differentiating non-LCSCs. These findings indicate that LCSCs have unique properties from those of non-LCSCs. Therefore, targeting the unique properties of LCSCs, such as oncogenes, oncoproteins, and essential signaling pathways, may offer an effective approach to killing LCSCs and preventing liver cancer metastasis.

#### Inducing differentiation of LCSCs and converting them into non-LCSCs

Cyclin D1 has been reported to play an important role in tumorigenesis and metastasis in multiple cancers, including HCC. Silencing of cyclin D1 decreases the percentage of CD133^+^ cells in HepG2 and SMMC-7721 cells, and overcomes 5-fluorouracil resistance^105^. In addition, because liver cancer cells are easily killed by anticancer drugs, inducing the differentiation of LCSCs and converting them into non-LCSCs may decrease the malignancy of liver cancer or inhibit its aggressiveness. Huang et al.^49^ have revealed that LCSCs release exosomes in a RAB27A-dependent manner, thus inducing Nanog expression and regorafenib resistance in differentiated cells. Targeting this exosome signaling pathway blocks the conversion of non-LCSCs to LCSCs.

#### Targeting the mechanical microenvironment of LCSCs in liver cancer tissue

Because the maintenance and distribution of LCSCs are affected by the mechanical heterogeneity of liver cancer tissue, and the stiffer invasion front margin facilitates the distribution and maintenance of stemness of LCSCs to a greater extent than the soft core^6^, it has the potential to destroy the mechanical microenvironment of LCSCs in liver cancer tissue, thereby damaging to the mechanical microenvironment that maintains LCSC stemness and function. Many *in vitro* experiments have revealed that a stiffer matrix is conducive to maintaining LCSC stemness. For example, Li et al.^69^ have cultured LCSCs in a 3D alginate matrix with different stiffness and found that a stiffer matrix (72.2 kPa), compared with a soft matrix (7.7 kPa), significantly potentiates LCSC stemness. Wei et al.^106^ have collected surgical specimens from patients with HCC and detected the cross-section stiffness at different sites (core: 1.051 kPa; middle: 4.54 kPa; margin: 9.307 kPa) with AFM. The authors have designed a polyacrylamide hydrogel with a stiffness of 1.10 kPa, 4.47 kPa, and 10.61 kPa to simulate the effect of mechanical heterogeneity on HCCLM3 and Huh7 cell lines, and have found that the mRNA and protein levels of NANOG and OCT4 of LCSC markers increase with increasing hydrogel stiffness. Zhao et al.^107^ have demonstrated that α2δ1^+^ HCC TICs initiate ECM remodeling by secreting LOX, thus creating a stiff microenvironment with crosslinked collagen, which favors the acquisition and subsequent maintenance of the stem cell-like properties of α2δ1^+^ TICs. Wang et al.^108^ have demonstrated that liver tumor tissues from metastatic patients have higher matrix stiffness than the non-metastatic tissues, and matrix stiffness equips tumor cells with enhanced stemness and proliferative characteristics dependent on the activation of the integrin β1/FAK/ERK1/2/NF-κB signaling pathway. Yang et al.^109^ have demonstrated that the cancer stem cell markers EpCAM, CD133, and ALDH-1 are elevated in Hep3B and Huh7 cells *via* the upregulation of CXCR4 expression, on stiff gels (12 kPa) compared with soft polyacrylamide hydrogels (1 kPa). These results indicate that the maintenance of LCSC stemness is affected by the mechanical cues of the matrix. Targeting the mechanical microenvironment of LCSCs in liver cancer tissues may provide a new strategy for the clinical treatment of liver cancer.

## Conclusion and perspectives

The development of liver cancer is closely associated with changes in the liver mechanical microenvironment, and LCSCs promote the progression and metastasis of liver cancer. However, several challenges remain. First, the roles of mechanical factors in the development of liver cancer are not well understood or investigated. Second, research is lacking on the mechanical heterogeneity of liver cancer tissue that regulates the enrichment of LCSCs and the mechanism of malignant metastasis of liver cancer. Third, normal stem cell biology largely influences the current understanding of LCSCs: the current understanding of LCSCs greatly relies on normal stem cells and LCSCs sharing many of the same expression markers and activating self-renewal signaling pathways. LCSCs are removed by targeting these markers and signaling pathways; however, this process may also lead to the removal of normal liver stem/progenitor cells, thereby inhibiting liver regeneration and resulting in liver failure. Consequently, further research is needed to identify markers specific to LCSCs. Finally, the mechanical heterogeneity of liver cancer tissues considerably affects the distribution and maintenance of LCSC stemness. Disrupting the mechanical microenvironment of LCSCs may damage the microenvironment that LCSCs rely on to maintain their stemness and exert their functions, thus causing them to differentiate into non-LCSCs, and blocking the malignancy and metastasis of liver cancer. However, no effective strategy is currently available for targeting LCSCs on the basis of the mechanical heterogeneity of liver cancer tissues.

In summary, the development of liver fibrosis, cirrhosis, and liver cancer and changes in the mechanical microenvironment of the liver tissue are closely associated with the development of liver cancer tissue. Furthermore, the distribution, stemness maintenance, and functions of LCSCs are associated with the mechanical heterogeneity of liver cancer tissues. Understanding the 2 key factors of the mechanical microenvironment and LCSCs in liver carcinogenesis may provide theoretical guidance for preventing and treating liver cancer in clinical settings.
